# Identification of Inhibitors of ZIKV Replication

**DOI:** 10.3390/v12091041

**Published:** 2020-09-18

**Authors:** Desarey Morales Vasquez, Jun-Gyu Park, Ginés Ávila-Pérez, Aitor Nogales, Juan Carlos de la Torre, Fernando Almazan, Luis Martinez-Sobrido

**Affiliations:** 1 Clinical and Translational Science Institute, University of Rochester School of Medicine and Dentistry, Rochester, New York, NY 14625, USA; dvasquez@txbiomed.org; 2 Department of Microbiology and Immunology, University of Rochester, Rochester, New York, NY 14625, USA; jpark@txbiomed.org (J.-G.P.); gines_ap@hotmail.com (G.Á.-P.); nogales.aitor@inia.es (A.N.); 3 Department of Disease Intervention and Prevention, Texas Biomedical Research Institute, San Antonio, TX 78227, USA; 4 Department of Immunology and Microbiology, The Scripps Research Institute, La Jolla, CA 92037, USA; juanct@scripps.edu; 5 Department of Molecular and Cell Biology, Centro Nacional de Biotecnología (CNB-11 CSIC), Universidad Autonόma de Madrid, 28049 Madrid, Spain; falmazan@cnb.csic.es

**Keywords:** flavivirus, Zika virus, antivirals, therapeutic, drug treatment, ReFRAME library, drug repurposing

## Abstract

Zika virus (ZIKV) was identified in 1947 in the Zika forest of Uganda and it has emerged recently as a global health threat, with recurring outbreaks and its associations with congenital microcephaly through maternal fetal transmission and Guillain-Barré syndrome. Currently, there are no United States (US) Food and Drug Administration (FDA)-approved vaccines or antivirals to treat ZIKV infections, which underscores an urgent medical need for the development of disease intervention strategies to treat ZIKV infection and associated disease. Drug repurposing offers various advantages over developing an entirely new drug by significantly reducing the timeline and resources required to advance a candidate antiviral into the clinic. Screening the ReFRAME library, we identified ten compounds with antiviral activity against the prototypic mammarenavirus lymphocytic choriomeningitis virus (LCMV). Moreover, we showed the ability of these ten compounds to inhibit influenza A and B virus infections, supporting their broad-spectrum antiviral activity. In this study, we further evaluated the broad-spectrum antiviral activity of the ten identified compounds by testing their activity against ZIKV. Among the ten compounds, Azaribine (SI-MTT = 146.29), AVN-944 (SI-MTT = 278.16), and Brequinar (SI-MTT = 157.42) showed potent anti-ZIKV activity in post-treatment therapeutic conditions. We also observed potent anti-ZIKV activity for Mycophenolate mofetil (SI-MTT = 20.51), Mycophenolic acid (SI-MTT = 36.33), and AVN-944 (SI-MTT = 24.51) in pre-treatment prophylactic conditions and potent co-treatment inhibitory activity for Obatoclax (SI-MTT = 60.58), Azaribine (SI-MTT = 91.51), and Mycophenolate mofetil (SI-MTT = 73.26) in co-treatment conditions. Importantly, the inhibitory effect of these compounds was strain independent, as they similarly inhibited ZIKV strains from both African and Asian/American lineages. Our results support the broad-spectrum antiviral activity of these ten compounds and suggest their use for the development of antiviral treatment options of ZIKV infection.

## 1. Introduction

Zika virus (ZIKV) is a mosquito-borne arbovirus transmitted by the mosquito species *Aedes* [1]. ZIKV is a member of the genus Flavivirus within the family *Flaviviridae*, closely related to Yellow Fever and Dengue viruses (YFV and DENV, respectively), West Nile virus (WNV), and Japanese encephalitis virus (JEV) [2]. The ZIKV genome is comprised of a 10.8 kb single-stranded positive-sense RNA molecule that contains a 5′ and 3′ untranslated region (UTR) and a single open reading frame (ORF). The ORF encodes for a large polyprotein that is co- and post-translationally cleaved into three structural (capsid, C; pre-membrane, prM; and envelope, E) and seven nonstructural (NS1, NS2A, NS2B, NS3, NS4A, NS4B, and NS5) proteins [3]. ZIKV was first discovered in 1947 in the Zika forest of Uganda during a YFV investigation, and a second virus isolation came from *Aedes africanus* in 1948 [4]. After its discovery, ZIKV infections were only sporadically reported in Asia and Africa until 2007 [5], when an outbreak of ZIKV occurred on Yap island, in the Federated States of Micronesia, where about 73% of the residents on the island were infected [6]. In 2013, an outbreak of ZIKV occurred in French Polynesia, where 88% of infected patients reported symptomatic infections, including the development of Guillain-Barré syndrome (GBS) [7]. In 2015, local transmission of ZIKV was reported in Latin America and the Caribbean, where ZIKV outbreaks were associated with congenital microcephaly through maternal infection, and a large increase in the number of Guillain-Barré syndrome cases [8]. By May 2019, ZIKV was spread to over 84 countries and is now a global health problem [9]. There is an urgent medical need for the development of prophylactic and therapeutic intervention strategies to control ZIKV infections due to its potential for re-emergence [10]. However, to date, there are currently no Food and Drug Administration (FDA)-approved vaccines (prophylactic) and/or antivirals (therapeutic) for the treatment of ZIKV infection.

ZIKV vaccine development has been very challenging due to existing cross-reactive anti-DENV antibodies that augment viral infections [11]. There are multiple ZIKV candidate vaccines under development with several of them in phase 2 clinical trials [12,13,14]. There have also been efforts for developing effective therapies against ZIKV [15], and a number of antivirals have been identified in vitro, but their safety and efficacy in vivo remain to be determined and none have been licensed for the treatment of ZIKV infection [9].

The process of discovery and implementation of antivirals is an extensive and complex process, which requires extensive testing for safety and efficacy. Drug repurposing offers several advantages over developing an entirely new drug for a given indication, as drug repurposing can drastically reduce the timeline and resources required to advance a candidate antiviral into the clinic. The Repurposing, Focused Rescue and Accelerated Medchem (ReFRAME) collection is comprised of about 12,000 compounds [16]. Our screen of the ReFRAME library identified ten compounds with activity against the mammarenavirus lymphocytic choriomeningitis (LCMV) [16]. The identified compounds included inhibitors of adenosine triphosphate (ATP) synthesis (Antimycin A), inhibitors of dihydroorotate dehydrogenase (DHODH) that is a key enzyme of the pyrimidine biosynthesis pathway (Brequinar), inhibitors of orotidine monophosphate decarboxylase (OMPD) which catalyzes key steps in pyrimidine synthesis (Azauridine, Azaribine, and Pyrazofurin), inhibitors of inosine monophosphate dehydrogenase (IMPDH) which inhibit replication of RNA and DNA via GTP reduction (AVN-944, Mycophenolic acid, and Mycophenolate mofetil), and regulators of apoptosis (OSU-03012 and Obatoclax). We have shown that these compounds with antiviral activity against LCMV, also had inhibitory activities against influenza A and B viruses [17], identifying these compounds as broad-spectrum antiviral candidates, which led us to examine their ability to inhibit ZIKV infection. The ten tested compounds had effective prophylactic and therapeutic activities against divergent strains of the African and Asian/American ZIKV lineages, further supporting their broad-spectrum antiviral activity. Our results suggest the feasibility of repurposing these compounds for the treatment of ZIKV infection.

## 2. Material and Methods

### 2.1. Cell Lines

African green monkey kidney epithelial Vero (ATCC, CCL-81, Manassas, VA, USA) and human adenocarcinoma alveolar basal epithelial A549 (ATCC, CCL-185) cells were maintained in Dulbecco’s modified Eagle’s medium (DMEM; Mediatech, Inc., Manassas, VA, USA) supplemented with 5% fetal bovine serum (FBS) and 1% PSG (100 U/mL penicillin, 100 µg/mL streptomycin, and 2 mM l-glutamine) at 37 °C in a 5% CO_2_ atmosphere.

### 2.2. ZIKV Strains

ZIKV Paraiba/2015 isolate was provided by Stephen Dewhurst (Department of Microbiology and Immunology, University of Rochester, Rochester, NY, USA). ZIKV Uganda/1947 (MR-766 strain) was obtained from the Biodefense and Emerging Infections Research Resources Repository (BEI Resources, NR-50065, Manassas, VA, USA). ZIKV Puerto Rico/2015 (PRVABC59 strain) was kindly provided by the Centers for Disease Control and Prevention (CDC, Atlanta, GA, USA). Virus stocks were propagated in Vero cells and titrated by plaque assay as previously described [18,19].

### 2.3. Compounds

Antimycin A (Sigma-Aldrich, Catalog No. A8674, St. Louis, MO, USA), OSU-03012 (AkSci, Catalog No. Y0267, Union City, CA, USA), Obatoclax mesylate (Obatoclax; AkSci, Catalog No. V2454, Union City, CA, USA), 2′,3′,5′-Triacetyl-6-azauridine (Azaribine; Sigma-Aldrich, Catalog No. T340057, St. Louis, MO, USA), 6-azauridine (Azauridine; Sigma-Aldrich, Catalog No. A1882, St. Louis, MO, USA), Pyrazofurin (Sigma-Aldrich, Catalog No. SLM1502, St. Louis, MO, USA), Mycophenolate mofetil (AkSci, Catalog No. J90063, Union City, CA, USA), Mycophenolic acid (AkSci Catalog No. E480, Union City, CA, USA), AVN-944 (ADOOQ Bio, Catalog No. A13652, Irvine, CA, USA), and Brequinar sodium salt hydrate (Brequinar; Sigma-Aldrich, Catalog No. SML0113, St. Louis, MO, USA) and were prepared at 10 mM stock solution in dimethyl sulfoxide (DMSO). Aurintricarboxylic acid (ATA; Sigma-Aldrich, Catalog No. A1895, St. Louis, MO, USA) was prepared at 100 mM stock solution in dimethyl sulfoxide (DMSO) and was used as a positive control in these assays [15]. All compound stocks were kept at −20 °C until experimental use. Each drug was diluted in DMEM media supplemented with 5% FBS and 1% PSG for cell viability assays and in infectious DMEM media supplemented with 2% FBS and 1% PSG for viral inhibition assays, where the maximum DMSO concentration was 0.1%. Media containing 0.1% DMSO was included in each of the experiments as vehicle control.

### 2.4. Cell Viability Assays

Vero cell viabilities were determined using MTT (CellTiter 96 Non-Radioactive Cell Proliferation assay, Promega, Madison, WI, USA) and cell proliferation were determined using XTT (Cell Viability and Proliferation, Sigma-Aldrich, St. Louis, MO, USA) assays, following the manufacturer’s instructions. Briefly, confluent monolayers of Vero cells (96-well plate format, 5.0 × 10^4^ cells/well, quadruplicates) were treated with 100 µL of DMEM supplemented with 5% FBS and 1% PSG containing serially diluted (3-fold dilutions, starting concentration of 1350 µM) compounds or 0.1% DMSO (vehicle control). Cells were incubated at 37 °C for 36 h and treated with either 15 µL of Dye Solution for the MTT assay or 50 µL of XTT labeling reagent for the XTT assay at 37 °C in a 5% CO_2_ atmosphere for 4 h. MTT assay plates were then treated with 100 µL of Stop Solution and stored in a dark space overnight. Next, cell absorbance of MTT and XTT assays were measured at 570 nm using a Vmax Kinetic Microplate Reader (Molecular Devices, Waltham, MA, USA). Viability of compound-treated cells was calculated as a percentage relative to values obtained with DMSO vehicle-treated cells. Non-linear regression curves and the median cytotoxic concentration (CC_50_) were calculated using GraphPad Prism software version 8.2.1.

### 2.5. Viral Inhibition Assays

For the post-infection assays, confluent monolayers of Vero or A549 cells (96-plate format, 5.0 × 10^4^ cells/well, quadruplicates) were infected with 25 plaque-forming units (PFU)/well of ZIKV Paraiba/2015 or MR-766 strains, or 50 PFU/well of PRVABC59 strain, for 2 h at 37 °C. The use of 50 PFU/well of PRVABC59 was due to differences of plaque size formation of ZIKV PRVABC59 as compared to ZIKV Paraiba/2015 and MR-766. After viral adsorption, cells were treated with 100 µL of infection media (DMEM supplemented with 2% FBS and 1% PSG) containing 3-fold dilutions (starting concentration of 100 µM) of the different compounds, or 0.1% DMSO vehicle control. For the pre-infection assay, Vero cells (96-plate format, 5.0 × 10^4^ cells/well, quadruplicates) were treated with the same dilutions of the different compounds or 0.1% DMSO vehicle control 12 h before virus infection. For the co-infection assays, viruses were incubated with the compounds or 0.1% DMSO vehicle control and used to infect confluent monolayers of Vero cells (96-plate format, 5.0 × 10^4^ cells/well, quadruplicates). In all cases, after viral absorption, virus inoculum was removed, and cells were washed with infection media before adding fresh infection media containing 1% Avicel (Sigma-Aldrich). Infected cells were incubated at 37 °C for 36 h for ZIKV Paraiba/2015 or 48 h for MR-766 and PRVABC59 strains. After viral infections, cells were fixed for immunostaining with 4% formaldehyde for 1.5 h at room temperature, the overlays removed, and the cells washed three times with 1x PBS. Cells were then permeabilized with 0.4% Triton X-100 in PBS for 15 min at room temperature. Then, plates were blocked with 2.5% bovine serum albumin (BSA) in PBS (blocking solution) for 1 h at 4 °C, followed by incubation with 1 µg/mL of the pan-flavivirus E protein monoclonal antibody (mAb) 4G2 (BEI resources; NR-50327, Manassas, VA, USA). Viral plaques were visualized using Vectastain ABC kit and DAB Peroxidase Substrate kit (Vector Laboratories Inc., Burlingame, CA, USA), following the manufacturer’s instructions. Stained plaques were analyzed using the CTL ImmunoSpot plate reader and counting software (Cellular Technology Limited, Cleveland, OH, USA). Individual wells from three independent experiments conducted in quadruplicates were used to calculate the average and standard deviation (SD) of viral inhibition using Microsoft Excel software. Virus titers were calculated as plaque-forming units (PFU)/mL. Non-linear regression curves and the half maximal effective concentration (EC_50_) were determined (GraphPad Software, San Diego, CA, USA, Version 8.2.1).

### 2.6. Growth Inhibition Assays

Confluent monolayers (24-well plate format, 2.5 × 10^5^ cells/well, triplicates) of Vero cells were infected at a multiplicity of infection (MOI) of 0.1 for 2 h at 37 °C with ZIKV Paraiba/2015 diluted in infection media. After viral absorption, cells were incubated with infection media containing the indicated concentrations (0.1, 1, and 10 EC_50_) of each compound, or 0.1% DMSO vehicle control (No drug). At 24, 48, and 72 h post-treatment tissue culture supernatants were collected and titrated on Vero cells by immunostaining (PFU/mL) [18,19].

### 2.7. Protection Against ZIKV-Induced Cytopathic Effect (CPE)

Confluent monolayers (24-well plate format, 2.5 × 10^5^ cells/well, triplicates) of Vero cells were infected with ZIKV Paraiba/2015 (MOI 0.1) and incubated with infection media containing the indicated concentrations (10, 1, 0.1, and 0 EC_50_) of each compound, as described above. At 48 h post-treatment images were obtained using an inverted fluorescence microscope (Nikon Eclipse TE2000, Nikon, Minato City, Tokyo, Japan).

### 2.8. Apoptosis Assay

Apoptosis levels were measured using the Caspase Glo 3/7 assay kit (Promega, Madison, WI, USA), following the manufacturers specifications. Briefly, confluent monolayers of Vero cells (24-well plate format, 2.5 × 10^5^ cells/well, triplicates) were mock-infected or infected (MOI 0.1) with the indicated viruses. After 2 h of virus absorption at 37 °C, the viral inoculum was removed and 500 µL of fresh viral post-infection media containing drugs at the indicated concentrations (0.1, 1, and 10 EC_50_) of each compound, or 0.1% DMSO vehicle control (No drug) were added. Post-infection media in the absence of drug was used as internal control in this assay. At 24, 48, and 72 h post-treatment, cells were harvested in the tissue culture supernatants and frozen at −80 °C until analysis. Cells lysates were incubated 1:1 with the Caspase-Glo 3/7 substrate in a 96-well plate in the dark for 1 h at room temperature, and luminescence at 570 nm was measured using a SpectraMax iD5 (Molecular Devices, Waltham, MA, USA) following manufacturer’s instructions.

### 2.9. Statistical Analysis

Data analysis was conducted using Prism software version 8.2.1 (GraphPad Software, San Diego, CA, USA, Version 8.2.1). The CC_50_ and EC_50_ were calculated using sigmoidal dose–response curves (GraphPad Software, San Diego, CA, USA, Version 8.2.1), and the selective index (SI) of each compound was evaluated by dividing the CC_50_ with the EC_50_. The unpaired Student’s t-test was used to evaluate significant differences. Data of three independent experiments in triplicates or quadruplicates are expressed as the mean ± standard deviation (SD) using Microsoft Excel software. Values were considered statistically significant when * *p* < 0.05, ** *p* < 0.01, *** *p* < 0.001; or no significance (n.s.).

## 3. Results

### 3.1. Antiviral Activity of Compounds Against ZIKV

**Figure 1:**
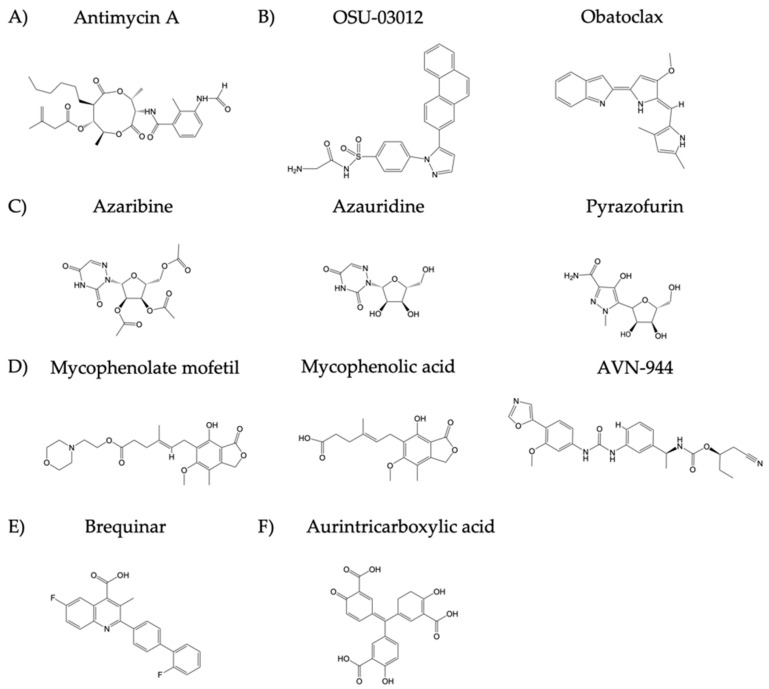
Chemical structure of the compounds: Groups of compounds include (**A**) inhibitors of adenosine triphosphate (ATP) synthesis (Antimycin A), (**B**) apoptosis inducers (OSU-03012 and Obatoclax), (**C**) inhibitors of orotidine monophosphate decarboxylase (OMPD), which catalyzes key steps in pyrimidine synthesis (Azaribine, Azauridine, and Pyrazofurin), (**D**) inhibitors of inosine monophosphate dehydrogenase (IMPDH) that inhibit replication of RNA and DNA via GTP reduction (Mycophenolate mofetil, Mycophenolic acid, and AVN-944), (**E**) inhibitors of dihydroorotate dehydrogenase (DHODH) that is a key enzyme of the pyrimidine biosynthesis pathway (Brequinar). (**F**) The JAK2 and STAT5 activator Aurintricarboxylic acid (ATA) was used as a positive control since it has been previously shown to have antiviral activity against ZIKV.

**Figure 2:**
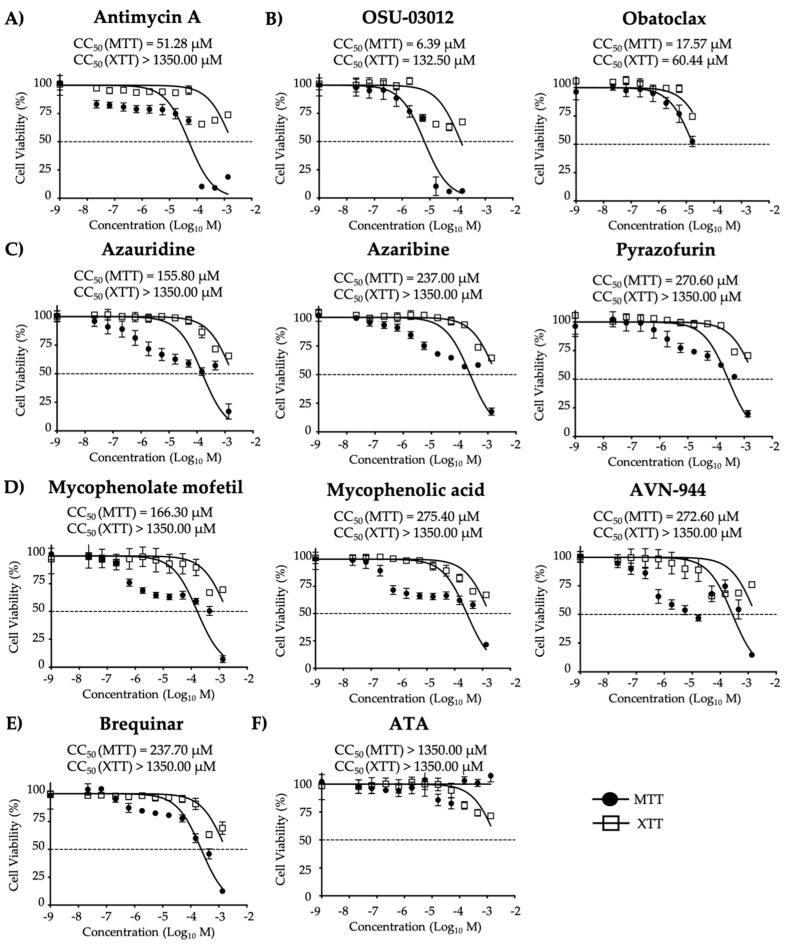
Cytotoxicity of the ten tested compounds: Vero cells (96-well plate format, 5 × 10^4^ cells/well, quadruplicates) were incubated with DMEM 5% FBS containing the indicated doses of the inhibitors (3-fold serial dilutions, starting concentration of 1350 µM) (**A**) Antimycin A, (**B**) OSU-03012 and Obatoclax, (**C**) Azaribine, Azauridine, and Pyrazofurin, (**D**) Mycophenolate mofetil, Mycophenolic acid, and AVN-944, (**E**) Brequinar, and (**F**) Aurintricarboxylic acid (ATA). Cell viability assays (MTT and XTT) were performed at 36 h post-treatment and the CC_50_ for each compound was calculated. Dotted line indicates the 50% toxicity of each of the compounds. Data were expressed as mean and SD from three independent experiments conducted in quadruplicates.

Prior to identifying which of the selected ten compounds, with antiviral activity against LCMV and influenza, (Figure 1) also had antiviral activity against ZIKV, we first evaluated their median cytotoxicity concentration (CC_50_), for each compound in non-infected Vero cells using cell viability (MTT) and cell proliferation (XTT) assays (Figure 2).

In the MTT assay, OSU-03012 (CC_50_-MTT = 6.39 µM) and Obatoclax (CC_50_-MTT = 17.57 µM) had the highest toxicity; Antimycin A (CC_50_-MTT = 51.28 µM), Azauridine (CC_50_-MTT = 155.80 µM), and Mycophenolate mofetil (CC_50_-MTT = 166.30 µM) had moderate toxicity; and Azaribine (CC_50_-MTT = 237.00 µM), Pyrazofurin (CC_50_-MTT = 270.60 µM), Mycophenolic acid (CC_50_-MTT = 275.40 µM), AVN-944 (CC_50_-MTT = 272.60 µM), and Brequinar (CC_50_-MTT = 237.70 µM) had the lowest toxicity (Figure 2 and Table 1). Results from the XTT assay showed no toxic effects even at the highest concentration tested (1350 µM) for each of the ten compounds except for OSU-03012 and Obatoclax (Figure 2 and Table 1). ATA, included as a control in these assays, showed a CC_50_ > 1350 µM in both, the MTT and the XTT assays, consistent with previously MTT CC_50_ published data (Figure 2 and Table 1) [15]. Due to pigment from compounds OSU-03012 and Obatoclax, higher concentration values were excluded because of the misrepresentation with the Vmax Kinetic Microplate Reader that impacted values based on absorbance. Using an MTT assay, we were able to show Azaribine, Pyrazofurin, Mycophenolic acid, AVN-944, and Brequinar had the least toxicity in Vero cells, and the XTT assay showed no toxic effects in all compounds, except OSU-03012 and Obatoclax. Similar results were observed for MTT and XTT at 72 h (Figure S1).

**Figure 3:**
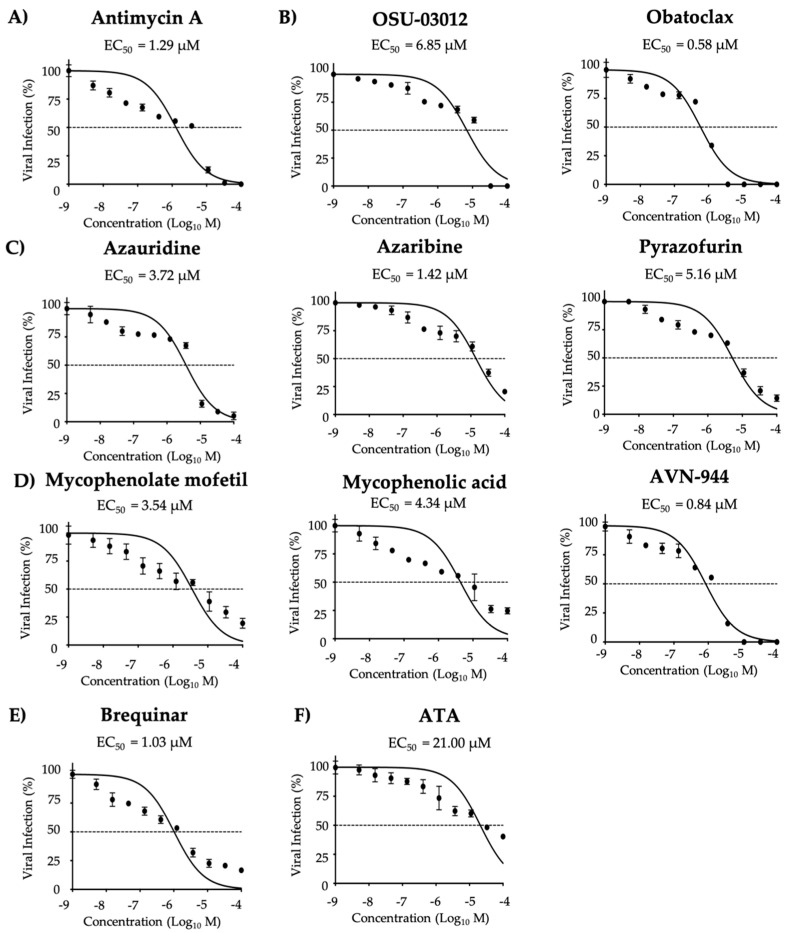
Inhibition of ZIKV Paraiba/2015 replication during post-treatment: Vero cells (96-well plate format, 5 × 10^4^ cells/well, quadruplicates) were infected with 25 PFU/well of Zika virus Paraiba/2015. After 2 h of viral absorption, media containing virus was replaced by infection media (DMEM 2% FBS) containing the indicated doses of the inhibitors (3-folds dilutions, starting concentration 100 µM) (**A**) Antimycin A, (**B**) OSU-03012 and Obatoclax, (**C**) Azaribine, Azauridine, and Pyrazofurin, (**D**) Mycophenolate mofetil, Mycophenolic acid, and AVN-944, (**E**) Brequinar, and (**F**) Aurintricarboxylic acid (ATA); and 1% Avicel. At 36 h post-treatment, the cells were fixed and immunoassayed using the anti-E 4G2 mAb. Plaques were counted with an automated ELISPOT reader. Dotted line indicates 50% inhibition. Data were expressed as mean and SD from three independent experiments conducted in quadruplicates.

We next determined the half maximal effective concentration (EC_50_) in post-treatment conditions (cells were infected for 2 h and then treated with the compounds) to evaluate the compounds potency, maximal effective concentrations were also evaluated (Table S1). Compounds potency to inhibit ZIKV multiplication was determined based on the EC_50_ and selectivity index (SI = CC_50_/EC_50_) values. Compounds were then categorized as potent, moderate to weak, and weak based on the SI value determined for each independent treatment condition. Inhibitory activity was observed for all ten compounds tested based on SI-MTT and SI-XTT values (Figure 3 and Table 1). Azaribine, AVN-944, and Brequinar were identified as the most potent inhibitors (Figure 3 and Table 1). Mycophenolate mofetil, Mycophenolic acid, Pyrazofurin, Azauridine, Obatoclax, and Antimycin A were identified as moderate to weak inhibitors (Figure 3 and Table 1). OSU-03012 was the weakest inhibitor in post-treatment conditions (Figure 3 and Table 1). ATA, included as a control in these assays, had a moderate to weak antiviral activity based on the SI-MTT and SI-XTT values, respectively (Figure 3 and Table 1). In post-treatment conditions, Azaribine, AVN-944, and Brequinar exhibited potent antiviral activity against ZIKV.

**Figure 4:**
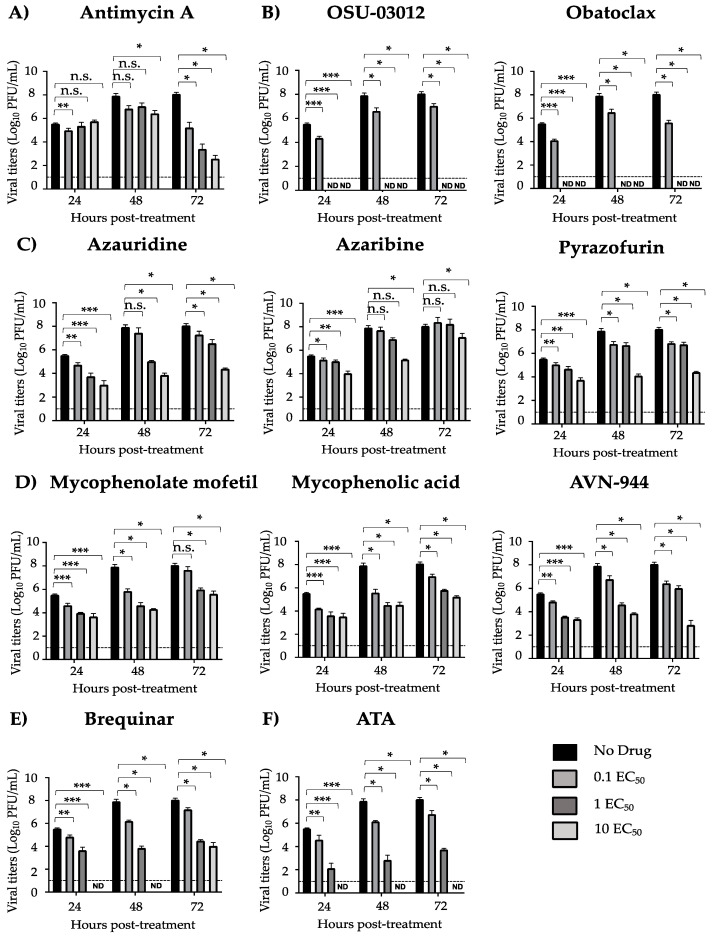
Growth kinetics inhibition of ZIKV Paraiba/2015: Vero cells (24-well plate format, 2.5 × 10^5^ cells/well, triplicates) were infected (MOI 0.1) with Paraiba/2015. After 2 h viral absorption, cells were treated with the indicated concentrations of compounds (0, 0.1, 1, and 10 EC_50_) (**A**) Antimycin A, (**B**) OSU-03012 and Obatoclax, (**C**) Azaribine, Azauridine, and Pyrazofurin, (**D**) Mycophenolate mofetil, Mycophenolic acid, and AVN-944, (**E**) Brequinar, and (**F**) Aurintricarboxylic acid (ATA); or 0.1% DMSO vehicle control (No drug) in infection media (DMEM 2% FBS). Tissue culture supernatants were collected at 24, 48, and 72 h post-treatment, and viral titers were calculated by immunostaining using the anti-E 4G2 mAb. Dotted line indicates the limit of detection (10 PFU). Data were expressed as mean and SD from three independent experiments conducted in triplicates. Statistical analysis was conducted by an unpaired Student’s t-test, * *p* < 0.05, ** *p* < 0.01, *** *p* < 0.001, or no significance (n.s.).

We next evaluated the effect of the ten compounds on production of infectious viral progeny in a growth kinetics inhibition assay (Figure 4). A dose-dependent inhibitory effect was observed for Azauridine, Pyrazofurin, Mycophenolate mofetil, Mycophenolic acid, AVN-944, and Brequinar at all time points (Figure 4). Likewise, a dose-dependent effect was observed for Antimycin A at 72 h post-treatment (Figure 4). A significant effect was observed for Azaribine at 24 h post-treatment and at 10 EC_50_ at 48 and 72 h post-treatment (Figure 4). Complete inhibition was observed at 1 EC_50_ for OSU-03012 and Obatoclax at all the analyzed times, which may have been caused by the inhibitory effect of the compound or by the toxicity of the compound, and at 10 EC_50_ for Brequinar at 24 and 48 h post-treatment (Figure 4). Consistent with previous findings, complete inhibition was observed at 10 EC_50_ for ATA (Figure 4) [15].

### 3.2. Pre- and Co-Treatment Effect of Compounds on ZIKV Infection

**Figure 5:**
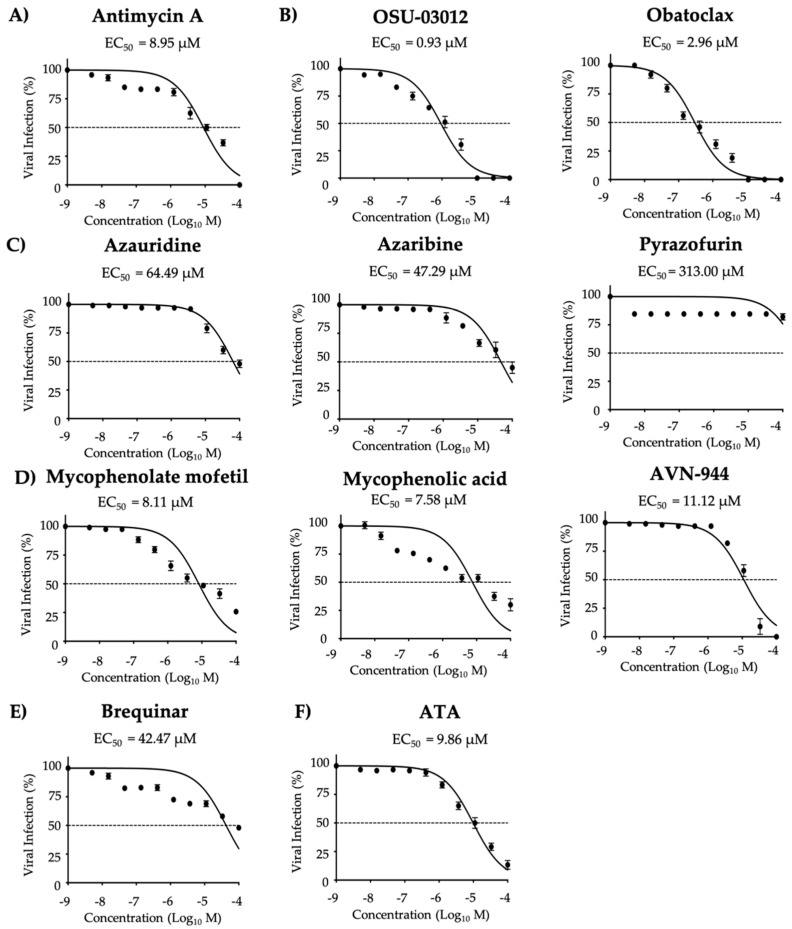
Inhibition of ZIKV Paraiba/2015 replication (pre-treatment): Vero cells in 96-well plate (5 × 10^4^ cells/well; quadruplicates) were treated with infection media (DMEM 2% FBS) containing the indicated concentrations of the compounds (3-folds dilutions, starting concentration 100 µM) (**A**) Antimycin A, (**B**) OSU-03012 and Obatoclax, (**C**) Azaribine, Azauridine, and Pyrazofurin, (**D**) Mycophenolate mofetil, Mycophenolic acid, and AVN-944, (**E**) Brequinar, and (**F**) Aurintricarboxylic acid (ATA). After 12 h of treatment, media was replaced, and cells were infected with Paraiba/2015 (25 PFU/well). After 2 h viral absorption, media was replaced by post-infection media. At 36 h post-infection, cells were fixed and immunoassayed using the anti-E 4G2 mAb. Plaques were counted with an automated ELISPOT reader. Dotted line indicates 50% inhibition. Data were expressed as mean and SD from three independent experiments conducted in quadruplicates.

**Figure 6:**
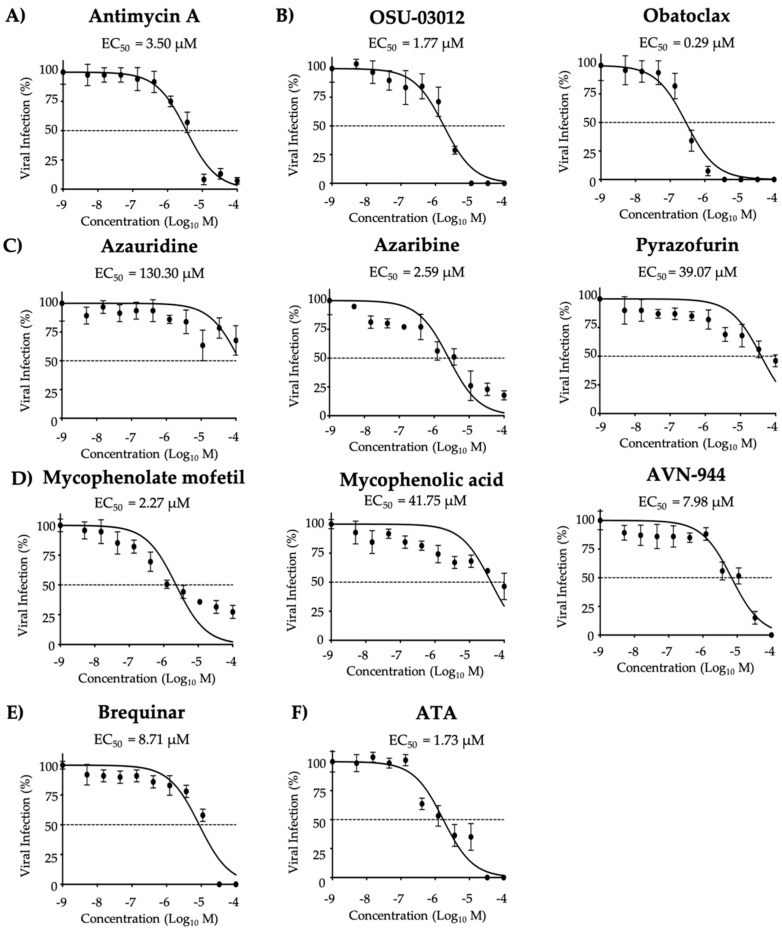
Inhibition of ZIKV Paraiba/2015 with the compounds (co-treatment): ZIKV Paraiba/2015 (25 PFU/well) were pre-incubated in infection media (DMEM 2% FBS) containing the indicated concentrations of the compounds (3-folds dilutions, starting concentration 100 µM) (**A**) Antimycin A, (**B**) OSU-03012 and Obatoclax, (**C**) Azaribine, Azauridine, and Pyrazofurin, (**D**) Mycophenolate mofetil, Mycophenolic acid, and AVN-944, (**E**) Brequinar, and (**F**) Aurintricarboxylic acid (ATA) for 1 h. Vero cells (96-well plates, 5 × 10^4^ cells/well; quadruplicates) were then infected with the virus-compound mixtures and after 2 h viral absorption, media was replaced by post-infection media. At 36 h post-infection, the cells were fixed and immunoassayed with the anti-E 4G2 mAb. Plaques were counted with an automated ELISPOT reader. Dotted line indicates 50% inhibition. Data were expressed as mean and SD from three independent experiments conducted in quadruplicates.

Next, we tested the prophylactic (pre-treatment) (Figure 5 and Table 2) and co-treatment inhibitory (Figure 6 and Table 3) activity of the ten compounds against ZIKV Paraiba/2015, maximal effective concentrations were also evaluated (Table S1). Potent prophylactic antiviral activity was observed for Mycophenolate mofetil, Mycophenolic acid, and AVN-944 based on SI-MTT values, whereas all other compounds were moderate to weak inhibitors (Figure 5 and Table 2). Based on SI-XTT values, OSU-03012 had the highest potent prophylactic antiviral activity, and Pyrazofurin, Azauridine, Azaribine, Obatoclax, and Brequinar demonstrated the weakest prophylactic antiviral activity (Figure 5 and Table 2). ATA, included as a control, exhibited potent and moderate prophylactic inhibitor based on the SI-MTT and SI-XTT values, respectively (Figure 5 and Table 2). In pre-treatment conditions, Mycophenolate mofetil, Mycophenolic acid, and AVN-944 exhibited potent antiviral activity against ZIKV based on SI-MTT and OSU-03012 exhibited potent antiviral activity based on SI-XTT.

Potent co-treatment inhibitory activity was observed for Obatoclax, Azaribine, and Mycophenolate mofetil based on SI-MTT values, while OSU-03012, Azauridine, Pyrazofurin, and Mycophenolic acid showed weak co-treatment inhibitory activity (Figure 6 and Table 3). Based on SI-XTT values, Antimycin A, Azaribine and Mycophenolate mofetil had potent prophylactic antiviral activity, whereas all other compounds were moderate to weak inhibitors (Figure 6 and Table 3). ATA, included as a control in these assays, was identified as a potent prophylactic inhibitor based on the SI-MTT and SI-XTT values (Figure 6 and Table 3). In co-treatment conditions, Obatoclax, Azaribine, and Mycophenolate mofetil exhibited potent antiviral activity against ZIKV based on MTT and based on XTT Antimycin A, Azaribine and Mycophenolate mofetil were the most potent among all the tested compounds.

### 3.3. Protection from ZIKV-Induced Cell Death

**Figure 7:**
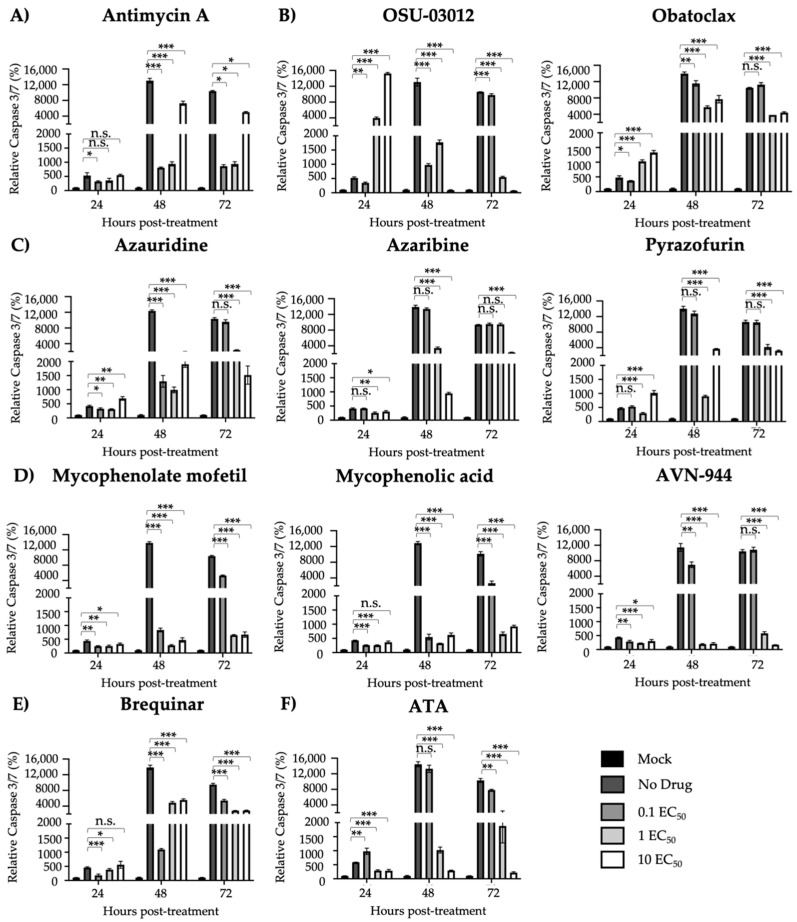
Prevention of apoptosis-induced cell death: Vero cells (24-well plate format, 2.5 × 10^5^ cells/well, triplicates) were infected (MOI 0.1) with Paraiba/2015. After 2 h viral absorption, infected cells were treated with the indicated concentrations of compounds (0, 0.1, 1, and 10 EC_50_) (**A**) Antimycin A, (**B**) OSU-03012 and Obatoclax, (**C**) Azaribine, Azauridine, and Pyrazofurin, (**D**) Mycophenolate mofetil, Mycophenolic acid, and AVN-944, (**E**) Brequinar, and (**F**) Aurintricarboxylic acid (ATA); and Caspase 3/7 levels were measured at 24, 48, and 72 h post-treatment. Data of each time point was compared to mock-infected control cells and expressed as mean of relative percentage and SD from three independent experiments conducted in triplicates. Statistical analysis was conducted by an unpaired Student’s *t*-test, * *p* < 0.05, ** *p* < 0.01, *** *p* < 0.001, or no significance (n.s.).

Next, we evaluated the ability of the compounds to prevent ZIKV-induced cell death, tissue culture supernatants and cells were harvested at 24, 48, and 72 h post-treatment to measure the activity of Caspase 3 and 7, key proteins in the apoptotic pathway (Figure 7). Low levels of caspase activation were observed for all compounds treated with 1 and 10 EC_50_, except for Obatoclax, Azaribine, Antimycin A, and Pyrazofurin (Figure 7). A dose-dependent effect was observed for ATA treated compounds as previously described (Figure 7F) [15]. All compounds apart from Obatoclax, Mycophenolic acid, and Antimycin A were able to prevent ZIKV-induced CPE. Similar results were observed with the Caspase 3/7 assay, where low levels were exhibited by all compounds except for Obatoclax, Azaribine, Antimycin A, and Pyrazofurin.

### 3.4. Effect of the Compounds on Old World and New World ZIKV Strains

**Figure 8:**
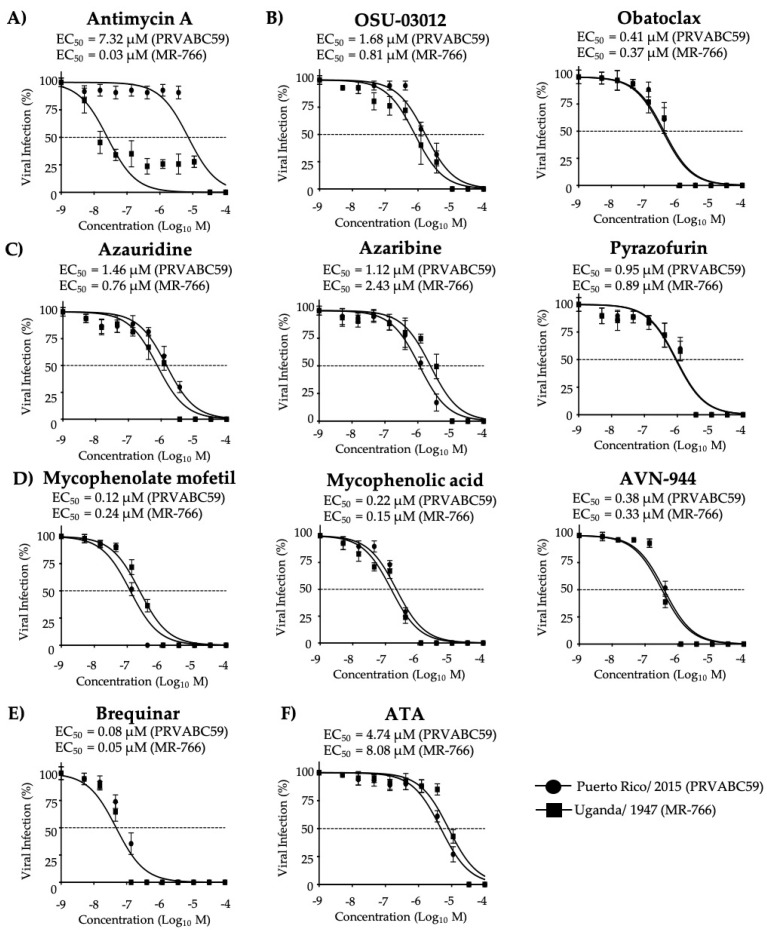
Inhibition of African (MR-766) and Asian/American (PRVABC59) prototype ZIKV strains: Vero cells (96-well plates, 5 × 10^4^ cells/well, quadruplicates) were infected with 25 PFU of MR-766, and 50 PFU of PRVABC59 ZIKV strains. After 2 h viral absorption, the indicated concentrations of compounds (3-folds dilutions, starting concentration 100 µM) (**A**) Antimycin A, (**B**) OSU-03012 and Obatoclax, (**C**) Azaribine, Azauridine, and Pyrazofurin, (**D**) Mycophenolate mofetil, Mycophenolic acid, and AVN-944, (**E**) Brequinar, and (**F**) Aurintricarboxylic acid (ATA) were added to post-infection media. Infected cells were fixed for virus titration by immunostaining assay at 36 h (MR-766) or 48 h (PRVABC59) post-treatment. Dotted line indicates 50% inhibition. Data were expressed as mean and SD from three independent experiments conducted in quadruplicates.

We next determined the ability of the compounds to inhibit representative old World (MR-766) and new World (PRVABC59) ZIKV strains to assess whether their antiviral activities were strain independent. Maximal effective concentrations were also evaluated (Table S2). In post-treatment conditions, all ten compounds tested exhibited inhibitory effects with the most potent inhibitors of ZIKV old World (MR-766) being Antimycin A, Brequinar, Mycophenolic acid, Mycophenolate mofetil, and AVN-944, based on both the SI-MTT and the SI-XTT values, whereas all other tested compounds were moderate to weak inhibitors (Figure 8 and Table 4). ATA, included as a control, exhibited a weak inhibitory effect based on the SI-MTT and SI-XTT values (Figure 8 and Table 4).

Based on the SI-MTT and SI-XTT values, Brequinar, Mycophenolate mofetil, Mycophenolic acid, and AVN-944 exhibited potent inhibitory effect on ZIKV new World strain PRVABC59. All other compounds exhibited moderate or weak inhibitory effects (Figure 8 and Table 5). ATA, included as a control, exhibited moderate (SI-MTT) or weak (SI-XTT) inhibitory activities (Figure 8 and Table 5).

### 3.5. Effect of the Compounds on ZIKV Infection in Human A549 Cells

**Figure 9:**
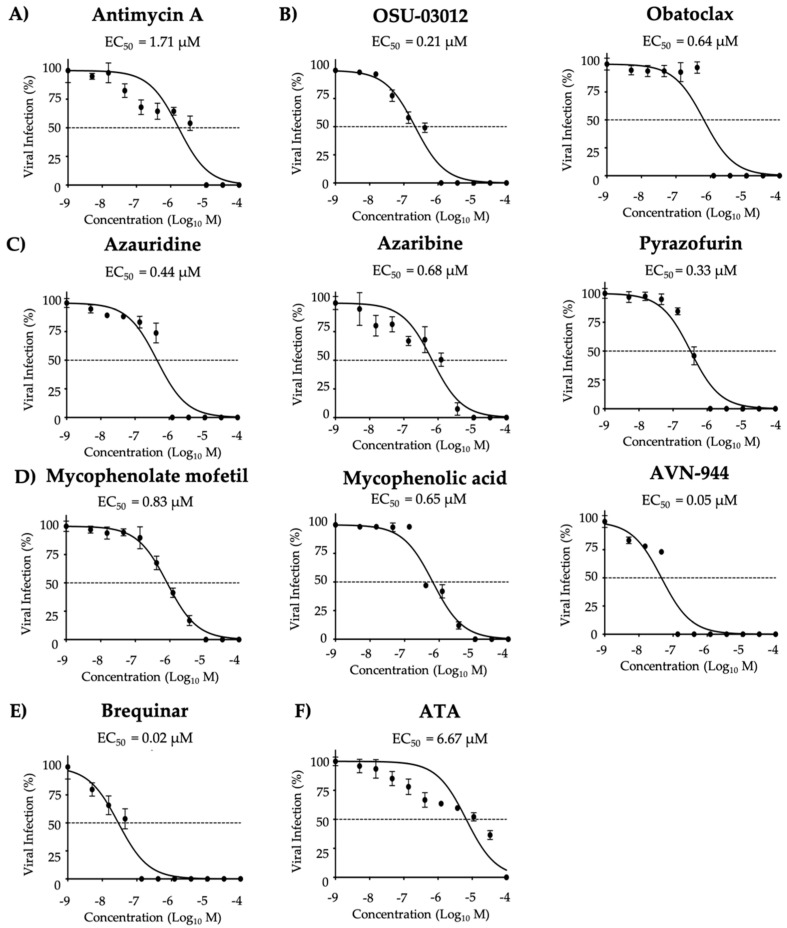
Inhibition of ZIKV replication in A549 cells: Human A549 cells in 96-well plate (5 × 10^4^ cells/well; quadruplicates) were infected (25 PFU/well) with Paraiba/2015. After 2 h viral absorption, post-infection media (DMEM 2% FBS) containing the indicated doses of the compounds (3-folds dilutions, starting concentration 100 µM) (**A**) Antimycin A, (**B**) OSU-03012 and Obatoclax, (**C**) Azaribine, Azauridine, and Pyrazofurin, (**D**) Mycophenolate mofetil, Mycophenolic acid, and AVN-944, (**E**) Brequinar, and (**F**) Aurintricarboxylic acid (ATA) were added. At 36 h post-treatment, infected cells were fixed and immunostained with the anti-E 4G2 mAb. Plaques were counted with an automated ELISPOT reader. Dotted line indicates 50% inhibition. Data were expressed as mean and SD from three independent experiments conducted in quadruplicates.

To further characterize the anti-ZIKV activity of the ten compounds and its translation into a potential treatment for ZIKV infection in humans, we evaluated their antiviral activity and the effects on cell viability (MTT) and proliferation (XTT) in human alveolar A549 cells. Maximal effective concentrations were also evaluated (Table S3). Results from the MTT and XTT assay showed no toxic effects even at the highest concentration used for the all ten compounds (50.00 µM) (Table 6) [17]. Potent antiviral activity was observed for Brequinar and AVN-944, based on the SI-MTT and the SI-XTT values (Figure 9 and Table 6). Moderate or weak antiviral activity was observed for all other tested compounds (Figure 9 and Table 6). ATA, included as a control in these assays, exhibited a weak inhibitory effect based on the SI-MTT and SI-XTT values (Figure 9 and Table 6).

## 4. Discussion

In this study, we investigated the anti-ZIKV activity of ten compounds from the ReFRAME library (Figure 1) that have been previously described as potent inhibitors of LCMV [16] and influenza virus [17]. These compounds included Antimycin A, which has been identified as a producer of reactive oxygen species (ROS); Azauridine, Azaribine, Pyrazofurin, Mycophenolate mofetil, Mycophenolic acid, AVN-944, and Brequinar identified to inhibit nucleoside biosynthesis; and Obatoclax and OSU-0312 identified to inhibit endosomal fusion. All compounds, except Obatoclax and Mycophenolic acid, exhibited antiviral activity against ZIKV infection in Vero and A549 cells that was unrelated to compound induced cell toxicity. Results from post-treatment conditions demonstrated values for SI-MTT ranging from 0.76 to 78.16 and SI-XTT ranging from 54.00 to 2142.86; pre-treatment conditions showed values ranging from 0.86 to 136.92 SI-MTT and 4.31 to 1451.61 SI-XTT; co-treatment conditions revealed SI-MTT values ranging from 1.19 to 780.34 and SI-XTT values ranging from 10.36 to 1655.17. We observed similar results when the compounds were tested against old and new World ZIKV strains, as well as in human A549 cells, supporting the repurposing of these compounds as broad-spectrum antivirals.

We identified the compounds as potent, moderate, or weak inhibitors of ZIKV based on their SI value, both MTT and XTT, calculated for each treatment condition, and grouped them based on their mechanism of action. Group A, consisting of Antimycin A, an inhibitor of mitochondrial electron transport chain (mETC) complex III, leading to the production of reactive oxygen species (ROS) [20]. Antimycin A has been previously described to have antiviral activity against various RNA viruses (Table 7) [17,21]. Results from using various conditions revealed Antimycin A to be a moderate inhibitor when used in pre- and co-treatment and a weak inhibitor when used in post-treatment based on SI-MTT; and a potent inhibitor in post- and co-treatment and a moderate inhibitor in pre-treatment based on SI-XTT. Antimycin A was a potent inhibitor and weak inhibitor of MR-766 and PRVABC59 ZIKV strains, respectively, based on both SI-MTT and SI-XTT (Table 4 and Table 5). In A549 cells, Antimycin A was identified as a weak inhibitor based on the SI-MTT and SI-XTT (Table 6). Despite its weak inhibitory effect in post-treatment assays, Antimycin A might still be of value for treatment of ZIKV infection due to its pre- and co-treatment antiviral activity.

Group B included the apoptosis inducers OSU-03012 and Obatoclax. OSU-03012 is described as a celecoxib derivative kinase inhibitor that does not inhibit cyclooxygenase activity, but instead downregulates the PI3K/Akt pathway that is involved in multiple cell signaling pathways [22]. OSU-03012 has exhibited in vitro and/or in vivo antimicrobial activities against a wide range of pathogens (Table 7) [22,23]. OSU-03012 has also been described to inhibit ZIKV PRVABC59 in Huh-7 cells (EC_50_ of 0.82–0.88 µM), U251 cells (EC_50_ of 0.84 µM) and SF268 cells (EC_50_ of 1.18 µM) [22], and against ZIKV-MEX-2-81 in U2OS cells (EC_50_ of 0.9 µM) [24]. Here, we have identified OSU-03012 as a weak post- and co- treatment inhibitor (Table 2 and Table 3, respectively) and a moderate pre-treatment inhibitor based on SI-MTT (Table 2), and a potent pre- and co-treatment inhibitor, and a weak post-treatment inhibitor based on SI-XTT. In addition, OSU-03012 was a weak inhibitor of ZIKV MR-766 based on SI-MTT and moderate inhibitor based on SI-XTT (Table 4), and a weak inhibitor of PRVABC59 based on SI-MTT and moderate inhibitor based on SI-XTT (Table 5). In A549 cells, OSU-03012 was identified as a moderate inhibitor based on the SI-MTT and SI-XTT (Table 6). Obatoclax has been described as a cell-directed anticancer compound that targets cellular myeloid leukemia cell differentiation protein (Mcl-1) and it has been shown to inhibit endocytosis and membrane fusion of various viruses (Table 7) [25,26]. Obatoclax has been previously described to have anti-ZIKV activity in retinal pigment epithelial (RPE) cells infected with ZIKV FB-GWUH-2016 (EC_50_ 0.04 µM) [27], but it did not exhibit inhibitory activity in JEG-3 cells infected with a ZIKV cocktail of MR-766, FSS13025, and MEX-2-81 [24]. We identified Obatoclax as a weak post-treatment inhibitor (Table 1), a moderate pre-treatment inhibitor (Table 2), and a potent co-treatment inhibitor (Table 3) based on SI-MTT values and as a potent post-, pre-, and co-treatment inhibitor based on SI-XTT values. Co-treatment results were consistent with the ability of Obatoclax to inhibit membrane fusion for other viruses. Obatoclax was a weak inhibitor of ZIKV MR-766 based on SI-MTT and moderate inhibitor based on SI-XTT (Table 4). In addition, it is a weak inhibitor of PRVABC59 based on SI-MTT and a potent inhibitor based on SI-XTT (Table 5). In A549 cells, Obatoclax was identified as a weak inhibitor based on the SI-MTT and SI-XTT (Table 6). Although not identified as a potent inhibitor in any condition, OSU-03012 has the ability to inhibit ZIKV replication for multiple strains. Obatoclax was identified as a potent co-treatment inhibitor making it a good candidate for the treatment of ZIKV infection.

Group C included Azaribine, Azauridine, and Pyrazofurin, which are known inhibitors of orotidine monophosphate decarboxylase (OMPD), a key enzyme involved in the pyrimidine biosynthesis pathway. Azaribine, the triacetate salt of Azauridine, was developed for the treatment of psoriasis and has been identified as an antiviral inhibitor of several RNA viruses (Table 7) [17,28,29]. Our results identified Azaribine as a potent post- and co-treatment inhibitor based on SI-MTT and SI-XTT (Table 1 and Table 3, respectively) and a moderate pre-treatment inhibitor based on SI-MTT and weak inhibitor based on SI-XTT (Table 2). Azaribine was a weak inhibitor of ZIKV MR-766 based on SI-MTT and SI-XTT (Table 4), and a moderate inhibitor of PRVABC59 based on SI-MTT and SI-XTT (Table 5). In A549 cells, Azaribine was identified as a weak inhibitor based on the SI-MTT and SI-XTT (Table 6). Azauridine inhibits de novo pyrimidine synthesis and DNA synthesis and is converted intracellularly into mono-, di-, and tri-phosphate derivatives that can be incorporated into RNA and inhibit protein synthesis [30]. Azauridine has been described as a broad-spectrum antiviral with activity against various viruses (Table 7) [16,17,29,31,32,33]. Azauridine has been previously described to inhibit ZIKV DAKAR B11514 (EC_50_ of 1.5 µM), MR-766 (EC_50_ of 0.98 µM) and PRVABC59 (EC_50_ of 0.61 µM) strains in Vero cells, and against ZIKV KX197192.1 strain (EC_50_ of 2.3 µM) in Huh7 cells [32,34]. We identified Azauridine as a weak in pre- and co-treatment inhibitor based on SI-MTT and SI-XTT (Table 2 and Table 3, respectively) and a moderate post-treatment inhibitor based on SI-MTT and SI-XTT (Table 1). In addition, Azauridine was found to be a moderate inhibitor of ZIKV MR-766 based on SI-MTT and SI-XTT (Table 4) and a moderate inhibitor of PRVABC59 based on SI-MTT and SI-XTT (Table 5). In A549 cells, Azauridine was identified as a moderate inhibitor based on the SI-MTT and SI-XTT (Table 6). Pyrazofurin has been shown to be a potent antiviral agent that inhibits multiplication of various viruses (Table 7) [17,29,35,36]. Our results identified Pyrazofurin as a weak pre- and co-treatment inhibitor based on SI-MTT and SI-XTT (Table 2 and Table 3, respectively) and a moderate and weak post-treatment inhibitor based on SI-MTT and SI-XTT, respectively (Table 1). Pyrazofurin was a moderate inhibitor of ZIKV MR-766) and PRVABC59 based on SI-MTT and SI-XTT (Table 4 and Table 5, respectively), and in A549 cells, it was identified as a moderate inhibitor based on the SI-MTT and SI-XTT (Table 6). Altogether, Azaribine was identified as a potent post- and co-treatment inhibitor making it of value for treatment of ZIKV infections. Despite not being identified as potent inhibitors in any condition, Azauridine and Pyrazofurin have the ability to inhibit replication of multiple ZIKV strains.

Group D included Mycophenolate mofetil, Mycophenolic acid, and AVN-944, known inhibitors of inosine monophosphate dehydrogenase (IMPDH), a key enzyme in the de novo synthesis of purines. Inhibition of IMPDH can deplete guanine nucleotide pools, followed by a decrease in DNA and RNA synthesis [37]. Mycophenolate mofetil is the prodrug of Mycophenolic acid. It is currently approved for the prevention of acute renal allograft rejection and it has been shown to be effective in the treatment of refractory rejection in renal, heart, and liver transplant recipients, and may have efficacy in the treatment of chronic rejection as well [38]. It has been also shown to have antiviral activity against various viruses (Table 7) [17,37,39,40]. Anti-ZIKV activity of Mycophenolate mofetil has been described in JEG-3 cells infected with a ZIKV cocktail of MR-766, FSS13025, and MEX-2-81 strains (EC50 of 0.76 µM) [24]. Our results identified Mycophenolate mofetil as a moderate and potent pre-treatment inhibitor based on SI-MTT and SI-XTT, respectively (Table 2), a potent co-treatment inhibitor based on SI-MTT and SI-XTT (Table 3), and a moderate post-treatment inhibitor based on SI-MTT and SI-XTT (Table 1). In addition, Mycophenolate mofetil was a potent inhibitor of ZIKV MR-766 and PRVABC59 based on SI-MTT and SI-XTT (Table 4 and Table 5, respectively), and in A549 cells it was identified as a weak inhibitor based on the SI-MTT and SI-XTT (Table 6). Mycophenolic acid depletes guanosine nucleotides in T and B lymphocytes and inhibits their proliferation, thereby suppressing cell-mediated immune responses and antibody formation. It also inhibits the glycosylation and expression of adhesion molecules, and the recruitment of lymphocytes and monocytes into sites of inflammation [40]. Mycophenolic acid is commonly used for the prevention of rejection in kidney and liver transplantation and has been shown to inhibit flavivirus infection in mammalian cells by preventing the synthesis and accumulation of viral RNA, to suppress DENV2 infection in mosquito midguts and dissemination to salivary glands when administered through a blood or sugar meal, and to inhibit ZIKV infection in both mosquito cells and adults [41,42]. In addition, Mycophenolic acid has been documented to have antiviral activity against many viruses (Table 7) [17,43,44,45,46,47]. Mycophenolic acid has previously been shown to exhibit anti-ZIKV activity in Vero cells infected with ZIKV SL1602 strain (EC_50_ of 0.42 µM); in Huh7, HeLa and HAEC cells infected with ZIKV MEX_I_7 strain (EC_50_ < 2 µM); in U2OS cells infected with ZIKV MEX-2-81 strain (EC_50_ of 0.4 µM); and in JEG-3 and HBME cells infected with a ZIKV cocktail of MR-766, FSS13025, and MEX-2-81 strains (EC_50_ of 0.1 µM) and inhibitory effects were described in Vero cells infected with ZIKV MR-766 (EC_50_ of 0.11) and PRVABC59 (EC_50_ 0.14 µM) [24,34,48,49]. Our results identified Mycophenolic acid as a moderate post-treatment inhibitor based on SI-MTT and SI-XTT (Table 1), a moderate and a potent pre-treatment inhibitor based on SI-MTT and SI-XTT, respectively (Table 2), and a weak co-treatment inhibitor based on SI-MTT and SI-XTT (Table 3). Mycophenolic acid was also a potent inhibitor of ZIKV MR-766 and PRVABC59 based on SI-MTT and SI-XTT (Table 4 and Table 5, respectively). Inhibition of ZIKV MR-766 and PRVABC59 by Mycophenolic acid treatment EC_50_ values determined in Vero cells were similar to previously reported data (Table 4 and Table 5, respectively) [34]. In A549 cells Mycophenolic acid exhibited weak inhibitory activity based on the SI-MTT and SI-XTT (Table 6). AVN-944 has been described to have anticancer properties as well as broad spectrum antiviral activity against various viruses (Table 7) [17,37,50,51]. We identified AVN-944 as a potent post-treatment inhibitor of ZIKV based on SI-MTT and SI-XTT (Table 1), a potent pre-treatment inhibitor based on SI-MTT and a moderate inhibitor based on SI-XTT (Table 2), and a moderate co-treatment inhibitor based on SI-MTT and SI-XTT (Table 3). AVN-944 was a potent inhibitor of ZIKV MR-766 and PRVABC59 strains based on SI-MTT and SI-XTT (Table 4 and Table 5, respectively). In A549 cells, it was identified as a potent inhibitor based on the SI-MTT and SI-XTT (Table 6). Overall, Mycophenolate mofetil was identified as a potent co-treatment inhibitor and against MR-766 and PRVABC59 ZIKV strains making it of value for treatment of ZIKV infections. Although it was not identified as a potent inhibitor in any condition, Mycophenolic acid has the ability to inhibit replication of multiple ZIKV strains. AVN-944 was identified as a potent post- and pre-treatment inhibitor, and a potent inhibitor against MR-766 and PRVABC59 strains making it of value for treatment of ZIKV infections.

Group E included Brequinar, a known inhibitor of dihydroorotate dehydrogenase (DHODH). Brequinar has been described to have broad-spectrum antiviral activity against a large number of viruses (Table 7) [52,53,54,55,56,57]. Brequinar has also been shown to have anti-ZIKV activity in Vero cells infected with ZIKV MR-766 and PRVABC59 (EC_50_ of 0.08 and 0.08 µM, respectively), and in Vero cells infected with ZIKV MR-766 in pre-treatment conditions (EC_50_ of 0.3 µM) [34,56]. In the present work, we identified Brequinar as a potent post-treatment inhibitor based on SI-MTT and SI-XTT (Table 1), a moderate pre-treatment inhibitor based on SI-MTT and a weak inhibitor based on SI-XTT (Table 2), and a moderate co-treatment inhibitor based on SI-MTT and SI-XTT (Table 3). Brequinar was also a potent inhibitor of ZIKV MR-766 and PRVABC59 based on SI-MTT and SI-XTT (Table 4 and Table 5, respectively). Inhibition of ZIKV MR-766 and PRVABC59 by Brequinar in Vero cells exhibited EC_50_ values similar to those previously reported (Table 4 and Table 5, respectively) [34]. In A549 cells, Brequinar was identified as a potent inhibitor based on the SI-MTT and SI-XTT (Table 6). Altogether, Brequinar was identified as a potent post-treatment inhibitor and a potent inhibitor against MR-766 and PRVABC59 strains making it of value for treatment of ZIKV infections.

**Table 7:** Antiviral activity of Antimycin A, OSU-03012, Obatoclax, Azaribine, Azauridine, Pyrazofurin, Mycophenolate mofetil, Mycophenolic acid, AVN-944, and Brequinar against other viruses.

Group	Compound	Literature Findings		References
A	Antimycin A	Viruses	*Togaviridae* (Venezuelan equine encephalitis virus, VEEV), *Bunyaviridae* (La Crosse virus, LACV), *Picornaviridae* (encephalomyocarditis virus, EMCV) *Rhabdoviridae* (vesicular stomatitis virus, VSV), *Paramyxoviridae* (Sendai virus, SeV), *Flaviviridae* (hepatitis C virus, HCV), *Orthomyxoviridae* (influenza A and B viruses) families	[17,21]
B	OSU-03012	Bacteria	*Salmonella enterica* and *Francisella tularensis*	[22,23]
		Fungi	*Candida albicans*, *Cryptococcus neoformans*, *Fusarium* sp., mucorales, *Blastomyces dermatitidis*, *Histoplasma capsulatum*, and *Coccidioides immitis*	
		Parasite	*Leishmania donovani*	
		Viruses	Lassa virus, LASV; Marburg virus, MARV; Ebola virus, EBOV; DENV; Junin virus, JUNV; rubella virus, RV; and human immunodeficiency virus, HIV	
	Obatoclax	Viruses	Influenza A and B viruses, Bunyamwera virus (BUNV), and Sindbis virus (SINV), Chikungunya virus (CHIKV) and Semliki Forest virus (SFV)	[25,26]
C	Azaribine	Viruses	WNV and influenza A and B viruses	[17,28,29]
	Azauridine	Viruses	CHIKV, SFV, YFV, DENV, JEV, LCMV, parainfluenza 3 virus (HPIV-3), polyomavirus (PV), influenza A and B viruses, and WNV	[16,17,29,31,32,33]
	Pyrazofurin	Viruses	Rhinovirus, Herpes simplex virus (HSV), vaccinia virus, WNV, *Picornaviridae* (polio and Coxsackie B4), SINV, YFV, *Paramyxoviridae* (measles virus, MV; and respiratory syncytial virus, RSV), *Orthomyxoviridae* (influenza A and B viruses), arenavirus (JUNV and Tacaribe virus, TCRV), and *Rhabdoviridae* (VSV)	[17,29,35,36]
D	Mycophenolate mofetil	Viruses	Influenza A and B viruses, foot-and-mouth disease virus (FMDV), and HPIV-2	[17,37,39,40]
	Mycophenolic acid	Viruses	Influenza A and B viruses, camelpox virus, cowpox virus, monkeypox virus, vaccinia virus, reovirus, and HCV, among	[17,43,44,45,46,47]
	AVN-944	Viruses	FDMV, influenza A and B viruses, and JUNV	[17,37,50,51].
E	Brequinar	Viruses	FMDV, HIV, DENV, WNV, YFV, Powassan virus (POW), Western equine encephalitis virus (WEEV), VSV, rotavirus, EBOV, and Cantagalo virus	[52,53,54,55,56,57]

In summary, our results show that the ten tested compounds are effective prophylactic, co-treatment inhibitors, and therapeutic broad-spectrum antiviral candidates, supporting considering their repurposing for the treatment of ZIKV infections. Due to the various mechanism of action exhibited by these compounds, they can be explored in combination therapy. The findings that the compounds inhibited different ZIKV strains, suggest they may be active against newly emerging strains.

## 5. Conclusions

Results suggest that repurposing the ten tested compounds and implementing them for the treatment of ZIKV infection would be an efficient approach towards the development of anti-ZIKV treatment options. Future in vivo, pharmacological, and toxicological studies need to be done to demonstrate the feasibility for their use in humans. Host-directed antivirals make drug repurposing and usage feasible as viruses mutate and evolve, contributing to the likelihood of developing resistance against treatments directed against the virus. The results from the ten tested ReFRAME compounds confirm the feasibility of using these broad-spectrum antivirals for treatment of ZIKV infection based on their strain independent capabilities and the results from multiple conditions tested.

## Figures and Tables

**Table 1:** CC_50_, EC_50_ and SI of the compounds on ZIKV Paraiba/2015 replication (post-infection).

Compound	CC_50_ (MTT) (µM)	CC_50_ (XTT) (µM)	EC_50_ (µM)	SI (MTT)	SI (XTT)
Antimycin A	51.28	>1350.00	2.00	25.64	>675.00
OSU-03012	6.39	132.50	8.40	0.76	15.77
Obatoclax	17.57	60.44	0.63	27.89	95.94
Azauridine	155.80	>1350.00	4.29	36.32	>314.69
Azaribine	237.00	>1350.00	1.62	146.29	>833.33
Pyrazofurin	270.60	>1350.00	5.96	45.35	>226.51
Mycophenolate mofetil	166.30	>1350.00	3.52	47.24	>383.52
Mycophenolic acid	275.40	>1350.00	4.26	64.65	>316.90
AVN-944	272.60	>1350.00	0.98	278.16	>1377.55
Brequinar	237.70	>1350.00	1.51	157.42	>894.04
ATA	>1350.00	>1350.00	25.00	>54.00	>54.00

CC_50_: median 50% cytotoxicity concentration; EC_50_: median 50% effective concentration; SI: selective index (CC_50_/EC_50_).

**Table 2:** CC_50_, EC_50_ and SI of the compounds on ZIKV Paraiba/2015 replication (pre-treatment).

Compound	CC_50_ (MTT) (µM)	CC_50_ (XTT) (µM)	EC_50_ (µM)	SI (MTT)	SI (XTT)
Antimycin A	51.28	>1350.00	8.95	5.73	>150.84
OSU-03012	6.39	132.50	0.93	6.87	142.47
Obatoclax	17.57	60.44	2.96	5.94	20.4
Azauridine	155.80	>1350.00	64.49	2.42	>20.93
Azaribine	237.00	>1350.00	47.29	5.01	>28.55
Pyrazofurin	270.60	>1350.00	313.00	0.86	>4.31
Mycophenolate mofetil	166.30	>1350.00	8.11	20.51	>166.46
Mycophenolic acid	275.40	>1350.00	7.58	36.33	>178.10
AVN-944	272.60	>1350.00	11.12	24.51	>121.40
Brequinar	237.70	>1350.00	42.47	5.59	>31.79
ATA	>1350.00	>1350.00	9.86	>136.92	>136.92

CC_50_: median 50% cytotoxicity concentration; EC_50_: median 50% effective concentration; SI: selective index (CC_50_/EC_50_).

**Table 3:** CC_50_, EC_50_ and SI of the compounds on ZIKV Paraiba/2015 replication (co-treatment).

Compound	CC_50_ (MTT) (µM)	CC_50_ (XTT) (µM)	EC_50_ (µM)	SI (MTT)	SI (XTT)
Antimycin A	51.28	>1350.00	3.50	14.65	>385.71
OSU-03012	6.39	132.50	1.77	3.61	36.70
Obatoclax	17.57	60.44	0.29	60.58	208.41
Azauridine	155.80	>1350.00	130.30	1.19	>10.36
Azaribine	237.00	>1350.00	2.59	91.51	>521.23
Pyrazofurin	270.60	>1350.00	39.07	6.93	>34.55
Mycophenolate mofetil	166.30	>1350.00	2.27	73.26	>594.71
Mycophenolic acid	275.40	>1350.00	41.75	6.59	>32.33
AVN-944	272.60	>1350.00	7.98	34.16	>169.17
Brequinar	237.70	>1350.00	8.71	27.29	>154.99
ATA	>1350.00	>1350.00	1.73	>780.34	>780.34

CC_50_: median 50% cytotoxicity concentration; EC_50_: median 50% effective concentration; SI: selective index (CC_50_/EC_50_).

**Table 4:** CC_50_, EC_50_ and SI of the compounds against old World ZIKV MR-766 strain.

Compound	CC_50_ (MTT) (µM)	CC_50_ (XTT) (µM)	EC_50_ (µM)	SI (MTT)	SI (XTT)
Antimycin A	51.28	>1350.00	0.03	1709.33	>45,000.00
OSU-03012	6.39	132.50	0.81	7.89	163.58
Obatoclax	17.57	60.44	0.37	47.49	163.35
Azauridine	155.80	>1350.00	0.76	205.00	>1776.32
Azaribine	237.00	>1350.00	2.43	97.53	>555.56
Pyrazofurin	270.60	>1350.00	0.89	304.04	>1516.85
Mycophenolate mofetil	166.30	>1350.00	0.24	692.92	>5625.00
Mycophenolic acid	275.40	>1350.00	0.15	1836.00	>9000.00
AVN-944	272.60	>1350.00	0.33	826.06	>4090.91
Brequinar	237.70	>1350.00	0.05	4754.00	>27,000.00
ATA	>1350.00	>1350.00	8.08	>167.08	>167.08

CC_50_: median 50% cytotoxicity concentration; EC_50_: median 50% effective concentration; SI: selective index (CC_50_/EC_50_).

**Table 5:** CC_50_, EC_50_ and SI of the compounds against new World ZIKV PRVABC59 strain.

Compound	CC_50_ (MTT) (µM)	CC_50_ (XTT) (µM)	EC_50_ (µM)	SI (MTT)	SI (XTT)
Antimycin A	51.28	>1350.00	7.32	7.01	>184.43
OSU-03012	6.39	132.50	1.68	3.80	78.87
Obatoclax	17.57	60.44	0.41	42.85	147.41
Azauridine	155.80	>1350.00	1.46	106.71	>924.66
Azaribine	237.00	>1350.00	1.12	211.61	>1205.36
Pyrazofurin	270.60	>1350.00	0.95	284.84	>1421.06
Mycophenolate mofetil	166.30	>1350.00	0.12	1385.83	>11,250.00
Mycophenolic acid	275.40	>1350.00	0.22	1251.82	>6136.36
AVN-944	272.60	>1350.00	0.38	717.37	>3552.63
Brequinar	237.70	>1350.00	0.08	2971.25	>16,875.00
ATA	>1350.00	>1350.00	4.74	>284.81	>284.81

CC_50_: median 50% cytotoxicity concentration; EC_50_: median 50% effective concentration; SI: selective index (CC_50_/EC_50_).

**Table 6:** CC_50_, EC_50_ and SI of the compounds against ZIKV Paraiba/2015 in A549 cells.

Compound	CC_50_ (MTT) (µM)	CC_50_ (XTT) (µM)	EC_50_ (µM)	SI (MTT)	SI (XTT)
Antimycin A	>50.00	>50.00	1.71 µM	>29.24	>29.24
OSU-03012	>50.00	>50.00	0.21 µM	>238.09	>238.09
Obatoclax	>50.00	>50.00	0.64 µM	>78.13	>78.13
Azauridine	>50.00	>50.00	0.44 µM	>113.64	>113.64
Azaribine	>50.00	>50.00	0.68 µM	>73.53	>73.53
Pyrazofurin	>50.00	>50.00	0.33 µM	>151.52	>151.52
Mycophenolate mofetil	>50.00	>50.00	0.83 µM	>60.24	>60.24
Mycophenolic acid	>50.00	>50.00	0.65 µM	>76.92	>76.92
AVN-944	>50.00	>50.00	0.05 µM	>1000.00	>1000.00
Brequinar	>50.00	>50.00	0.02 µM	>2500.00	>2500.00
ATA	>50.00	>50.00	6.67 µM	>7.49	>7.49

CC_50_: median 50% cytotoxicity concentration; EC_50_: median 50% effective concentration; SI: selective index (CC_50_/EC_50_).

## Supplementary Materials

The following are available online at https://www.mdpi.com/1999-4915/12/9/1041/s1, Figure S1: Cytotoxicity of the ten tested compounds, Table S1: Maximum effective concentration effects of the compounds post-, co-, and pre-treatment in Vero cells, Table S2: Maximum effective concentration effects of the compounds with old World ZIKV MR-766 strain and new World ZIKV PRVABC59, Table S3: Maximum effective concentration effects of the compounds in A549 cells.
