# Supplementary material for: Identification of Inhibitors of ZIKV Replication
Source: Viruses. 2020 Sep 18;12(9):1041. doi: 10.3390/v12091041 (PMC7551609; doi:10.3390/v12091041)
Supplement: Supplementary file 1 [file viruses-12-01041-s001.zip › viruses-904328-supplementary.pdf]

viruses

MDPI

Supplementary Materials: Identification of Inhibitors of ZIKV Replication

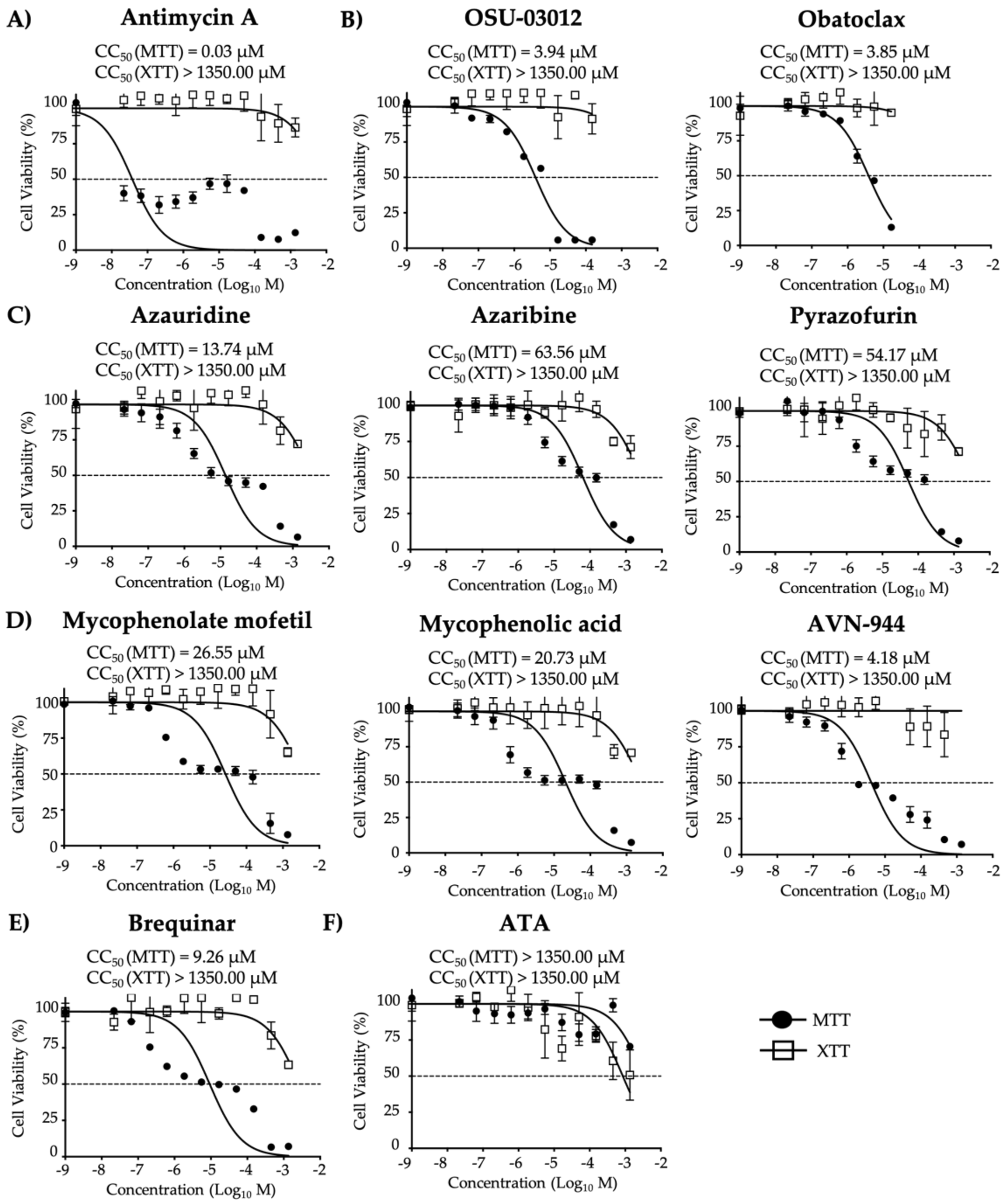

Figure S1. Cytotoxicity of the ten tested compounds: Vero cells (96-well plate format, 5 × 10^4 cells/well, quadruplicates) were incubated with DMEM 5% FBS containing the indicated doses of the inhibitors (3-fold serial dilutions, starting concentration of 1350 µM) (**A**) Antimycin A, (**B**) OSU-03012 and Obatoclax, (**C**) Azaribine, Azauridine, and Pyrazofurin, (**D**) Mycophenolate mofetil, Mycophenolic acid, and AVN-944, (**E**) Brequinar, and (**F**) Aurintricarboxylic acid (ATA). Cell viability assays (MTT and XTT) were performed at 72 h post-treatment and the CC_{50} for each compound was calculated. Dotted line indicates the 50% toxicity of each of the compounds. Data was expressed as mean and SD from three independent experiments conducted in quadruplicates.

Table S1. Maximum effective concentration effects of the compounds post-, co-, and pre-treatment in Vero cells. MIC: maximum effective concentration.

Compound	Post-treatment MIC (μM)	Co-treatment MIC (μM)	Pre-treatment MIC (μM)
Antimycin A	<100.00	>100.00	<100.00
OSU-03012	<33.30	>100.00	<3.70
Obatoclax	<0.01	<3.70	<11.10
Azauridine	>100.00	<100.00	>100.00
Azaribine	>100.00	>100.00	>100.00
Pyrazofurin	>100.00	>100.00	>100.00
Mycophenolate mofetil	>100.00	>100.00	>100.00
Mycophenolic acid	>100.00	<11.10	>100.00
AVN-944	<11.10	>100.00	<100.00
Brequinar	>100.00	<33.30	>100.00
ATA	>100.00	<33.30	>100.00

Table S2. Maximum effective concentration effects of the compounds with old World ZIKV MR-766 strain and new World ZIKV PRVABC59. MIC: maximum effective concentration.

Compound	MR766 MIC (μM)	PRVABC59 MIC (μM)
Antimycin A	<33.30	<33.30
OSU-03012	<11.10	>11.10
Obatoclax	<1.23	<1.23
Azauridine	<3.70	<11.10
Azaribine	<11.10	<11.10
Pyrazofurin	<3.70	<3.70
Mycophenolate mofetil	<1.23	<1.23
Mycophenolic acid	<1.23	<1.23
AVN-944	<1.23	<1.23
Brequinar	<0.13	<0.41
ATA	<33.30	<33.30

Table S3. Maximum effective concentration effects of the compounds in A549 cells. MIC: maximum effective concentration.

Compound	A549 cells MIC (μM)
Antimycin A	<11.10
OSU-03012	<1.23
Obatoclax	<1.23
Azauridine	<1.23
Azaribine	<11.10
Pyrazofurin	<1.23
Mycophenolate mofetil	<11.10
Mycophenolic acid	<11.10
AVN-944	<0.41
Brequinar	<0.04
ATA	<100.00
